# Is There a Genetic Relationship Between Alcoholism and Depression?

**Published:** 2002

**Authors:** John I. Nurnberger, Tatiana Foroud, Leah Flury, Eric T. Meyer, Ryan Wiegand

**Affiliations:** John I. Nurnberger, Jr., M.D., Ph.D., is the Joyce and Iver Small Professor of Psychiatry and professor of medical and molecular genetics; Tatiana Foroud, Ph.D., is an associate professor of medical and molecular genetics; and Leah Flury, M.S., is a biostatistician, all in the Department of Medical and Molecular Genetics, Indiana University School of Medicine, Indianapolis, Indiana. Eric T. Meyer, M.A., is information technology coordinator and Ryan Wiegand, M.S., is a biostatistician, both at the Institute of Psychiatric Research, Department of Psychiatry, Indiana School of Medicine, Indianapolis, Indiana

**Keywords:** genetic theory of AODU (alcohol and other drug use), AOD dependence, genetic trait, major depression, mood and affect disturbance, comorbidity, phenotype, chromosome, AODR (alcohol and other drug related) genetic markers, prevalence, gender differences, genetic linkage

## Abstract

The Collaborative Study on the Genetics of Alcoholism (COGA) seeks to identify genes contributing to alcoholism and related traits (i.e., phenotypes), including depression. Among alcoholic subjects the COGA study found an increased prevalence of depressive syndrome (i.e., depression that may or may not occur in conjunction with increased drinking). This combination of alcoholism and depression tends to run in families. Comorbid alcoholism and depression occurred substantially more often in first-degree relatives of COGA participants with alcoholism than in relatives of control participants. Based on these data, COGA investigators defined three phenotypes—“alcoholism,” “alcoholism and depression,” and “alcoholism or depression”—and analyzed whether these phenotypes were linked to specific chromosomal regions. These analyses found that the “alcoholism or depression” phenotype showed significant evidence for genetic linkage to an area on chromosome 1. This suggests that a gene or genes on chromosome 1 may predispose some people to alcoholism and others to depression (which may be alcohol induced).

It is obvious to drinkers that a direct connection exists between alcohol consumption and mood. Alcoholic intoxication commonly produces a “high” with attendant giddiness and lowering of inhibitions. Conversely, hangovers and acute withdrawal typically produce dysphoria, with elements of anxiety and depression mixed with physical malaise. Psychopathological studies have observed that alcoholism and affective disorders (e.g., depression and mania) interact and can coexist; moreover, the vulnerability to both alcoholism and depression can run in families (Merikangas and Gelernter 1990; Merikangas et al. 1994).

Various possible relationships between alcoholism and affective disorders have been postulated (see table 1) (for more information, see Nurnberger and Berrettini 1998; Merikangas and Gelernter 1990). For instance, some patients may use alcohol as a form of self-medication for an affective disorder. In these cases, alcoholism may develop secondarily to the affective disorder. Alternatively, depression may develop as a result of alcoholism; in these cases, alcoholism is the primary disorder and depression is considered an organic mood disorder (i.e., a mood disorder with a physiological cause). Other alternatives are that both alcoholism and affective disorder may develop as the result of a common genetic predisposition or may develop as completely separate illnesses. These different hypotheses about the relationship between alcoholism and affective disorders have different implications for the prevalence of these illnesses in family studies (see table 1). For example, if alcoholism were the primary disorder and depression occurred as a result of it, relatives of alcoholics would be expected to have an increased risk of alcoholism with secondary depression but not of depression alone. Relatives of people with depression but without alcoholism would be expected to have an increased risk of depression only. However, if depression were the primary disorder and alcoholism occurred secondarily to it, relatives of nondepressed alcoholics would be expected to have an increased risk of alcoholism only, whereas relatives of people with depression would be expected to have an increased risk of depression with secondary alcoholism.

Family data, such as those discussed later in this article, so far have not been entirely consistent with any single hypothesis but have suggested that several mechanisms may contribute to the relationship between alcoholism and affective disorders. Nevertheless, such analyses have confirmed that an association between alcoholism and affective disorders exists and that this association is at least partly mediated by genetic factors. The Genetics Initiative of the National Institute of Mental Health (NIMH) found that among families selected for multiple cases of bipolar disorder, males with major affective disorder had nearly twice the risk for alcoholism compared with males without affective disorder. Among females, the risk was raised sevenfold (Nurnberger and the NIMH Genetics Initiative Bipolar Group 2001). Kendler and colleagues (1993), who studied the co-occurrence of alcoholism and major depression in female twins, found a substantial genetic correlation (correlation coefficient of 0.4 to 0.6) between the two disorders.

Winokur and colleagues (1971) postulated that depressive illness could be divided into four types, depending on familial pattern of illness. These included (1) sporadic depressive disorder, which was nonfamilial; (2) pure depressive disorder, in which depression (but not alcoholism or sociopathy) was found in several relatives; (3) depressive spectrum disorder, in which depression as well as alcoholism or sociopathy was found in relatives of depressed subjects; and (4) bipolar depression, which was found in families with bipolar illness. It is of interest that the families examined in the Collaborative Study on the Genetics of Alcoholism (COGA), which is described in detail below, show an increased risk for sociopathy as well; interactions between depression and alcoholism, including the role of sociopathy, will be the subject of future analyses (Nurnberger et al. 2002). Investigators found various clinical distinctions among patients with these different types of depression. Perhaps the most impressive data were gathered by Schlesser and colleagues (1979), who found that people with pure depressive disorder showed a different pattern of activity of the stress hormone system (i.e., the hypothalamic–pituitary–adrenal system) than did people with depressive spectrum disorder or sporadic depression.

Thus, it appears likely that both alcoholism and depression exist in various forms (i.e., are heterogeneous) and that the co-occurrence (i.e., comorbidity) of both disorders may have different underlying mechanisms as well. Findings with animal models that have examined alcohol consumption and “depressive” behavior have also been heterogeneous. (For more information on such animal models, see the sidebar.) At this point, it is difficult to identify subtypes of both disorders on the basis of clinical criteria alone. Genetic studies such as COGA, however, may help with this distinction.

**Evidence of Co-Occurring Alcoholism and Depression in Animal Models**

The potential link between depression and alcohol use also has been investigated in laboratory animals. Although depression in animals cannot be assessed the same way as in humans, some behavioral tests can be interpreted as representing counterparts of human depression. Examples of these tests are the “Porsolt” or forced swim test, in which rats or mice are observed for the duration of their attempt to escape from a beaker of water, and the restrained stress test, a measure of the animals’ locomotor activity following a period of restraint in a plastic tube. In both of these tests, the animals exhibit greater activity when they are pretreated with antidepressant drugs.

Researchers have compared the results of behavioral tests for depression with voluntary alcohol consumption in defined strains of rodents. The results demonstrate variability in these animal models, similar to what is observed in human patients with depression and alcohol use disorders. For example, a rat strain called Flinders sensitive rat (FSL), which is thought to be particularly vulnerable to behavioral depression, does not voluntarily consume alcohol (see table) (Overstreet et al. 1992). Conversely, Fawn-hooded rats (Rezvani et al. 2002) and C57 mice (Elmer et al. 1987), both of which also demonstrate behavioral depression, will drink alcohol. Finally, P rats, which have been selectively bred for alcohol preference over many generations, do not respond to tests of behavioral depression (Godfrey et al. 1997).

Researchers also investigated the responses of these various animal strains to drugs that act on messenger chemicals implicated in alcohol’s effects on the brain. These analyses found that animals that are sensitive to depression also are sensitive to drugs which are similar to the messenger chemical but show variable responses to drugs that affect the messenger chemical (for a review, see Rezvani et al. 2002). These findings lead to the conclusion that, as in humans, animal models for both alcohol intake and depression show variability and that the relationship between the two behaviors varies with the model used.

—*John I. Nurnberger, Jr., Tatiana Foroud, Leah Flury, Eric T. Meyer, Ryan Wiegand*

**Table:** Relationship Between Behavioral Depression, as Indicated by Behavior in the “Forced Swim” and “Stress–Open Field” Tests, and Voluntary Alcohol Consumption in Genetically Defined Rodent Strains

Strain	Forced Swim Test	Stress–Open Field Test	Voluntary Alcohol Consumption
Flinders sensitive rat	**?**	**+**	**0**
P rat	**0**	−−	**++**
Fawn-hooded rat	**?**	**+**	**+**
C57 mouse	**++**	**+**	**+**

KEY: ? = response unknown

+ = sensitive to behavioral depression; voluntary alcohol consumption ++ = very sensitive to behavioral depression; high levels of voluntary alcohol consumption

0 = no sensitivity to behavioral depression; no voluntary alcohol consumption − = not tested

ReferencesElmerGIMeischRAGeorgeFRMouse strain differences in operant self-administration of ethanolBehavior Genetics1754394511987342650110.1007/BF01073111GodfreyCDFroehlichJCStewartRBComparison of rats selectively bred for high and low ethanol intake in a forced-swim-test model of depression: Effects of desipraminePhysiology and Behavior627297331997928449110.1016/s0031-9384(97)00171-6OverstreetDHRezvaniAHJanowskyDSGenetic animal models of depression and ethanol preference provide support for cholinergic and serotonergic involvement in depression and alcoholismBiological Psychiatry319199361992138625710.1016/0006-3223(92)90118-jRezvaniAHParsianAOverstreetDHThe Fawn-hooded (FH/Wjd) rat: A genetic animal model of comorbid depression and alcoholismPsychiatric Genetics12111620021190135410.1097/00041444-200203000-00001

The COGA project, conducted at several research centers across the United States, seeks to identify genes contributing to the development of alcoholism and related characteristics (i.e., phenotypes). This article describes some of the methods used by COGA investigators to define phenotypes related to the comorbidity of alcoholism and depression and summarizes data on both disorders in the COGA participants. Finally, the article discusses the implications of these findings for a potential genetic relationship between alcoholism and depression.

## Design and Methods of the COGA Study

Between 1988 and 1998, investigators at six COGA sites used a common protocol to gather clinical information and biological data (including DNA and neurophysiologic measures) from families of subjects with alcoholism. Participants were recruited among patients undergoing alcoholism treatment (i.e., the probands) and their first-degree relatives. Control families were recruited from dental clinics, motor vehicle records, or random mailings at the six sites. Control families were not excluded if a family member had alcoholism or another psychiatric disorder; thus, these families represent a comparison group not selected with respect to psychopathology. Each control family included at least two parents and three children ages 14 and older. All participants were interviewed using a screening instrument called the Semi-Structured Assessment for the Genetics of Alcoholism (SSAGA) (Bucholz et al. 1994), which allows for diagnostic assessment of various disorders, including alcoholism and depression.

For the analyses presented here, participants were diagnosed with alcoholism (ALC) if they met the diagnostic criteria for alcohol dependence specified in the *Diagnostic and Statistical Manual of Mental Disorders, Third Edition, Revised* (DSM–III–R) (American Psychiatric Association [APA] 1987) as well as the criteria for “definite alcoholism” established by Feighner and colleagues (1972). Participants were diagnosed with depression (DEP) if they met the DSM–III–R criteria for major depressive disorder or if they had “depressive syndrome.” (Subjects were classified as having depressive syndrome if they met all the criteria for major depressive disorder, except that the depression could have been caused by alcohol or other drug use or another illness.) Participants with both ALC and DEP were included in the phenotype “alcoholism and depression” (AAD). People meeting the criteria for either ALC or DEP were combined into a phenotype called “alcoholism or depression” (AorD). Separate analyses were conducted for participants with the DEP phenotype—that is, with depression or depressive syndrome (which can occur in both alcoholics and nonalcoholics). The COGA researchers performed statistical analyses of differences in the prevalence of alcoholism and/or depression in various subgroups of study participants and tested interactions between variables. These data were calculated for all families of alcoholic probands and for control families where appropriate.

After the diagnostic assessment, a subset of families with at least two alcoholic members in addition to the initially recruited proband were invited to participate in a second stage of assessment, which included blood collection for genetic analyses. For these analyses, the investigators checked a total of 336 short, repeated DNA sequences located throughout all chromosomes (for more information, see Reich et al. 1998; Nurnberger et al. 2001). These sequences are useful as markers because they vary in size from one person to another and their inheritance pattern can therefore be easily determined. This screening process was carried out with two groups of participants. The first group (the “initial data set”) included 987 people from 105 families, and the second group (the “replication data set”) included 1,295 people from 157 families.

To investigate the molecular genetics of alcoholism and depression, the COGA investigators performed linkage analyses. This means that they compared the presence of certain variants (i.e., alleles) of the markers in people with the ALC, AAD, AorD, and DEP phenotypes to identify chromosomal regions that were more similar in people with a given phenotype than would be expected by chance. Such regions would be considered genetically “linked” to the phenotype—that is, they are located near a gene that influences the phenotype. Because many genes appear to contribute to the risk for developing alcoholism, the investigators employed statistical methods that do not rely on specific models of susceptibility for the phenotype. All these statistical methods are based on the sharing of gene sequences that are identical by descent (IBD). Such sequences are considered IBD if both members of a sibling pair have inherited the sequence from the same parent. (For further discussion of linkage analysis for complex disorders, see Nurnberger and Berrettini 1998.) The investigators conducted multipoint linkage analyses, in which multiple markers were evaluated simultaneously for evidence of allele (gene sequence) sharing using the computer program ASPEX (ftp://lahmed.stanford.edu/pub/aspex/index.html).

## Results of the COGA Study

### Prevalence of Alcoholism and/or Depression

The COGA researchers first determined the prevalence of major depression and depressive syndrome in the families of the alcoholic probands (see table 2). These studies found that among both males and females, major depression was not more common in alcoholic participants than in nonalcoholic participants. However, depressive syndrome was significantly more common among male and female alcoholics than among nonalcoholics. As a result, the prevalence of the DEP phenotype (which combines both major depression and depressive syndrome) was increased in both alcoholic males and alcoholic females.

The combination of alcohol dependence and depression (i.e., the AAD phenotype) appears to run in families, as demonstrated by an analysis of first-degree relatives of alcoholic probands with or without depression and first-degree relatives of control subjects. This analysis found that AAD occurred in 15.9 percent of first-degree relatives of probands with AAD (489 out of 3,069 people), compared with 11.7 percent of first-degree relatives of probands with alcoholism alone (287 out of 2,462) and 3.6 percent of first-degree relatives of control subjects (42 out of 1,164). Thus, the prevalence of AAD was significantly greater among the first-degree relatives of probands with AAD than among relatives of probands with alcoholism alone or relatives of control subjects (Nurnberger et al. 2001).

Next, the investigators determined the risks of alcoholism or depression for the relatives of three types of alcoholic probands—those with alcoholism alone, those with alcoholism and depressive syndrome, and those with alcoholism and major depression. The risk of alcoholism was significantly increased in the relatives of probands with both types of depression compared with probands with alcoholism alone (see table 3). Similarly, the risk of depression was increased in relatives of alcoholic probands with major depression and, to a lesser extent, in relatives of alcoholic probands with depressive syndrome. These findings support the idea that the AAD phenotype may represent a genetically distinct condition.

This notion is further supported by the finding that depression in relatives of probands with AAD typically does not occur independently but only in combination with alcoholism. That is, the prevalence of major depression alone is not increased in those relatives. The prevalence of AAD, however, is increased twofold in relatives of probands with alcoholism plus depressive syndrome (i.e., 4.4 percent) and increased nearly fourfold in relatives of probands with alcoholism plus major depression (i.e., 8.4 percent) when compared with relatives of control subjects (i.e., 2.2 percent [see table 4]). Another analysis (not shown on the table) found that the prevalence of depression alone is not significantly increased in relatives of probands with alcoholism alone (19.6 percent) or with the AAD phenotype (21.2 percent) compared with relatives of control subjects (19.3 percent). The prevalence of the AAD phenotype, however, is increased in relatives of probands with alcoholism only (10.2 percent) and in relatives of probands with AAD (14.3 percent) compared with relatives of control subjects (3.4 percent). These findings argue for a model in which some families carry susceptibility factors for both conditions. Finally, the increase in the prevalence of the AAD phenotype was seen in both male and female relatives of probands with alcoholism only or AAD.

The researchers also explored the order in which alcoholism or depression developed in both the probands and their relatives. For this purpose, the investigators determined the ages of onset of alcoholism and depression, which according to the DSM–III–R are defined as the ages at which three symptoms of alcoholism or the first major depressive episode, respectively, occurred. The analyses found that in approximately 50 percent of subjects with AAD, the onset of major depression occurred prior to the onset of alcohol dependence (see table 5). (For comparison, mania occurred first in about 42 percent of subjects with both mania and alcoholism.) This finding may indicate that even in this group of families with multiple cases of alcohol dependence, a substantial number of people develop alcoholism secondary to an underlying mood disorder. However, there was a notable gender effect in the order of disease onset: Males tend to develop alcohol dependence before the onset of the affective disorder, whereas this order tended to be reversed in females.

### Linkage Analyses

Because the data presented in the previous section suggested some interaction of vulnerability factors for alcoholism and depression, the COGA investigators performed genetic linkage analyses using DNA samples from sibling pairs with the AAD, AorD, and DEP phenotypes in order to identify chromosomal regions linked to these phenotypes. In the sibling pairs, both siblings had the phenotype under investigation. This analysis included 224 AAD pairs (57 percent male), 1,359 AorD pairs (56 percent male), and 440 DEP pairs (49 percent male). The AorD phenotype is the most inclusive because it refers to people with either the ALC or DEP phenotypes. Most of the sibling pairs added to the ALC data set to generate the AorD data set (59 percent of all added pairs and 94 percent of the mixed gender pairs) consisted of an alcoholic brother with a depressed sister.

The results pointed to an area of interest on chromosome 1 for the AorD phenotype (Nurnberger et al. 2001). Increased allele sharing was seen near two markers called D1S1648 and D1S1588 between 100 and 110 centi-Morgan (cM)^1^ from the origin. This increased sharing was observed in the initial data set, to a lesser extent in the replication data set, and was still evident when the two data sets were combined. Overall, the analyses found evidence for genetic linkage over a relatively large portion of chromosome 1 (i.e., 60 cM). (For a summary of other linkage results from these data sets, including results for the AAD and DEP phenotypes, see Nurnberger and colleagues 2001; see also the articles in this issue by Bierut and colleagues, pp. 208–213 and by Edenberg, pp. 214–218.)

The same portion of chromosome 1 that exhibited linkage with the AorD phenotype also has shown suggestive linkage with the ALC phenotype (Reich et al. 1998). Analysis of all possible sibling pairs with the ALC phenotype in the initial data set identified a region near a marker called D1S1675. In sibling pairs with the ALC phenotype, allele sharing in that area was similar to the allele sharing observed in sibling pairs with the AorD phenotype. In these families, the same genetic characteristics may predispose some people to depression and others to alcoholism.

## Implications of the Study Results

The COGA study supports the conclusion of other investigators (Merikangas and Gelernter 1990; Merikangas et al. 1994) that alcoholism and depression tend to occur together and that comorbid alcoholism tends to aggregate in the relatives of probands with both disorders. The definition of depression in this analysis includes both major depression (i.e., primary depression) and depressive syndrome, which may be caused by alcohol and other drug use (i.e., secondary depression). However, primary and secondary depressive syndromes may not truly be distinct. Many people with alcohol problems spend a substantial portion of their lives drinking and thus have less opportunity to demonstrate independent episodes of depression. An alcoholic with true vulnerability for depression may, by the natural course of the two illnesses, have no demonstrably independent episodes.

The genetic analyses demonstrated evidence for linkage of the AorD phenotype with a region on chromosome 1, and the same region also showed evidence, though less substantial, of linkage with the ALC phenotype. This chromosome 1 region also showed possible linkage with mania and depression among the participants in the NIMH Genetics Initiative Bipolar Study (Rice et al. 1997). Preliminary results suggested that this finding may be accounted for by families in which the probands have both alcoholism and mania. Thus, although the interpretation of linkage results in complex diseases is the subject of ongoing controversy and must be done cautiously, it appears likely that a locus on chromosome 1 accounts for some of the familial aggregation of alcoholism and depression in the COGA study. The findings suggest that this region contains one or more genes associated with different clinical phenotypes (e.g., alcoholism, depression, and mania), a phenomenon called pleiotropy.

The gene or genes associated with these phenotypes have not yet been identified. However, several genes that may influence central nervous system function have been mapped to that region on chromosome 1. (For more information on genes located in that region, which is also referred to as the 1p13–35 region, see the Web site www.ncbi.nlm.nih.gov/locuslink.)

Researchers await the results of other studies that may confirm these findings. However, replication of linkage findings in complex disorders such as alcoholism and depression is likely to be difficult (Suarez et al. 1994). In disorders to which multiple genetic factors contribute but for which no single factor is absolutely necessary, evidence for specific effects can differ among different data sets (i.e., for different study samples). Accordingly, the replication of any given effect may require several studies with data sets of a size equivalent to the original data set.

The majority of participants with alcoholism in the COGA data set were male, and the majority of participants with depression were female. Of the sibling pairs added to the data set of alcoholic sibling pairs for the linkage analyses for the AorD phenotype, many consisted of a brother with alcoholism and a sister with depression. Consequently, the gene or genes contributing to these vulnerabilities may have variable expression in men and women. This result is reminiscent of the concept of depressive spectrum disease as postulated by Winokur and colleagues (1971).

The findings summarized in this article suggest a genetic relationship between depression and alcohol dependence in some families where both disorders are transmitted. This conclusion is consistent with the idea that depression can be caused by many different genes (i.e., is a genetically heterogeneous condition). The results obtained so far have no direct implications for the treatment of patients with depression and/or alcohol dependence. However, they do reinforce the idea that some heavy drinkers may have genetic vulnerability to depression, as well as the observation that treatment of depressed alcoholic patients with antidepressants has generally had beneficial effects on the depression, and sometimes on the drinking as well (McGrath et al. 2000). In the future, genetic studies are likely to contribute to clinical treatment by identifying specific genes and their biochemical pathways, which could result in new therapeutic options for patient subgroups.

The major advantage of the COGA study is its multisite design with similar methods employed at each site, which allowed the investigators to generate very large data sets. One limitation of the study is that by design it focused on families densely affected with alcohol dependence for linkage analysis (although all families of alcoholic probands are included in the prevalence studies). Although such families are ideal for genetic studies, they may not be fully representative of the spectrum of people who suffer from alcoholism, depression, or both. Despite this limitation, the study’s results, in combination with prior studies, suggest that the pattern of disorders in the family is a reasonable clinical characteristic to use for the differentiation of subgroups within alcoholism.

## Figures and Tables

**Table 1:** Hypotheses About the Relationship Between Alcoholism and Affective Disorders (e.g., Depression)

Then:	Relative risk of affective disorder in alcoholics	Relative risk of alcoholism in affective patients	Relatives of alcoholics would be at increased risk for:	Relatives of affective patients would be at increased risk for:
If it is hypo thesized that:				
Alcoholism is the primary disorder and affective disorder is secondary	Increases	Is unchanged	Alcoholism with secondary affective disorder	Affective disorder only
The affective disorder is primary and alcoholism is secondary	Is unchanged	Increases	Alcoholism only	Affective disorder with secondary alcoholism
Alcoholism and affective disorders develop from a common genetic predisposition	Increases	Increases	Both alcoholism and affective disorder	Both alcoholism and affective disorder
Genetic Subtype I (depressive spectrum disorder)	Is unchanged	Is unchanged	Alcoholism only	Both alcoholism and affective disorder
Genetic Subtype II (alcoholism with depression)	Increases, specifically for depressive syndrome	Is unchanged	Both alcoholism and depression	Affective disorder only
Alcoholism and affective disorder develop as separate illnesses	Is unchanged	Is unchanged	Alcoholism only	Affective disorder only

**Table 2:** Prevalence of DSM–III–R Depression and Depressive Syndrome in Adult COGA Probands and Their Alcoholic and Nonalcoholic Relatives

	COGA Probands and their Alcoholic Relatives	Nonalcoholic Relatives	Relative Risk Among COGA Probands and their Alcoholic Relatives
**Males**			
Major Depression	11.0% (257/2,337)	10.5% (151/1,436)	1.05
Depressive Syndrome	30.3%^a^ (707/2,337)	6.0% (86/1,436)	5.05
Total	41.2%^a^ (964/2,337)	16.5% (237/1,436)	2.50
**Females**			
Major Depression	24.2%^b^ (311/1,288)	22.4%^c^ (704/3,138)	1.08
Depressive Syndrome	32.8%^a^ (423/1,288)	11.3%^c^ (356/3,138)	2.90
Total	57.0%^b^ (734/1,288)	33.8%^c^ (1,060/3,138)	1.69

a χ^2^ ≥ 204 (actual values 315.4, 250.9, 290.9, 204.0, from top to bottom), *df* = 1, *p* < 10^−45^ vs. nonalcoholic subjects.

b χ^2^ ≥ 82 (actual values 108.6, 82.6), *df* = 1, *p* < 10^−18^ vs. males.

c χ^2^ ≥ 32 (actual values 92.1, 32.4, 144.7), *df* = 1, *p* < 10^−7^ vs. males.

NOTE: The data are from COGA Master File 86 (1999).

**Table 3:** Prevalence and Risk of Alcoholism and Depression in the Relatives of Alcoholic Probands and Control Subjects in the COGA Study

	Diagnosis in Relatives			
	
Proband Diagnosis	All Types of Alcoholism	Relative Risk of Alcoholism	All Types of Depression	Relative Risk of Depression
Control	14.2% (144/1,014)		20.9% (212/1,014)	
Alcoholism Only	29.2% (971/3,331)	2.1	28.6% (952/3,331)	1.4
Alcoholism with Depressive Syndrome	31.6%* (1,059/3,357)	2.2	33.5%* (1,123/3,357)	1.6
Alcoholism with Major Depression	32.8%* (306/934)	2.3	39.0%† (364/934)	1.9

* *p* < .05 vs. alcoholism only

† *p* < .05 vs. alcoholism with depressive syndrome All comparisons significant vs. control

NOTE: The data were derived from COGA Master file 86 (1999).

**Table 4:** Prevalence of Alcoholism and Depression in the Relatives of Alcoholic and Control Probands in the COGA Study

	Diagnosis in Relatives		
	
Proband Diagnosis	Alcoholism with or without Depressive Syndrome	Major Depression Only	Alcoholism and Major Depression
Control	10.2% (74/725)	14.6% (106/725)	2.2% (16/725)
Alcoholism with or without Depressive Syndrome	25.6%^a^ (1,112/4,348)	13.1% (571/4,348)	4.4%^a^ (191/4,348)
Alcoholism with Major Depression	24.0%^a^ (132/549)	14.2% (78/549)	8.4%† (46/549)

* *p* < .01 vs. control

† *p* < .001 vs. alcoholism only and control

NOTE: The data were derived from COGA Master File 86 (1999).

**Table 5:** Chronological Order of Disease Development in COGA Participants with Alcoholism and an Affective Disorder (i.e., Major Depression or Mania)

	Onset of Alcoholism First	Onset of Depression First	Same Time of Onset for Both Disorders
**Alcoholism and Depression**			
Males (N = 267)	143 (53.6%)	104 (39.0%)	20 (7.5%)
Females (N = 325)	115 (35.4%)	193 (59.4%)	17 (5.2%)
Total (N = 592)	258 (43.6%)	297 (50.2%)	37 (6.3%)
**Alcoholism and Mania**			
Males (N = 33)	22 (66.7%)	9 (23.7%)	2 (6.1%)
Females (N = 33)	13 (39.4%)	19 (57.6%)	1 (3.0%)
Total (N = 66)	35 (53.0%)	28 (42.4%)	3 (4.5%)

NOTE: The diagnoses and age of onset are based on data from COGA Master File 118 (2002).

## Footnotes

1 A centi-Morgan is a unit of measurement for distances along chromosomes. The largest human chromosome, chromosome 1, has a length of approximately 325 centi-Morgan. One can also express the location of markers as their distance in centi-Morgan from the tip of the chromosome.
